# Quality of Life as Medicine: A Pilot Studyof Patients with Chronic Illness and Pain

**DOI:** 10.1100/tsw.2003.36

**Published:** 2003-06-16

**Authors:** Soren Ventegodt, Joav Merrick, JØrgen Anderson

**Affiliations:** ^1^ The Quality of Life Research Center and Research Clinic for Holistic Medicine, Teglgårdstræde 4, DK-1452 Copenhagen K, Denmark; ^2^ National Institute of Child Health and Human Development, Division of Community Health, Zusman Child Development Center, Ben Gurion University, Beer-Sheva and Office of the Medical Director, Division for Mental Retardation, Ministry of Social Affairs, Jerusalem, Israel; ^3^ Norwegian School of Management, Norway and the foundation Stiftelsen Holistisk Medisin Scandinavia, Norway

**Keywords:** quality of life, QoL, screening, questionnaires, SEQOL, chronic pain, chronic illness, human development, Denmark

## Abstract

An intensive 5-day quality-of-life (QoL) session was constructed based on a psychosomatic model. The session was comprised of teaching on philosophy of life, psychotherapy, and body therapy. The three elements were put together in such a way that they mutually supported each other. The synergy attained was considerable. The pilot study demonstrated that in the course of only 1 week, patients had time to revise essential life-denying views and to integrate important, unfinished life events involving negative feelings. Consequently, the patients became more present in the body’s blocked-off areas and subjectively healthier. Nineteen persons with chronic illness and pain (fibromyalgia, chronic tiredness, whiplash, mild depression, and problems involving pain in arms and legs including osteoarthritis), and unemployed for 5–7 years attended the course. In the week before and after the 5-day course, the participants completed the validated SEQOL (Self-Evaluation of Quality of Life Questionnaire) including questions on self-evaluated health and the unvalidated “Self-Evaluation of Working-Life Quality Questionnaire” (SEQWL). This pilot study was without a control group or clinical control. As far as diagnoses were concerned, the group was inhomogeneous. Common for the group was a low QoL, poor quality of working life QWL, and numerous health problems. The study showed an 11.2% improvement in QoL (*p* < 0.05), a 6.3% improvement in QWL (*p* < 0.05), and a 12.0% improvement in self-perceived physical health (*p* = 0.08). There was a 17.3% improvement in self-perceived psychological health (*p* < 0.05) and satisfaction with health in general improved by 21.4% (*p* < 0.05). Symptoms like pain were almost halved and several of the participants were free of pain for the first time in years. In conclusion it seemed that the combination of training in philosophy of life, psychotherapy, and body therapy can give patients a large, fast, and efficient improvement in QoL, QWL, and health. It is not known if these changes will be permanent and if all kinds of patients with different health problems will gain from this cure. Further research should be conducted.

## INTRODUCTION

Since the 1990s, the Quality-of-Life Research Center in Copenhagen has studied the connection between quality of life (QoL) and illness with more than 1,000 factors influencing the QoL analysed to identify the factors most important and convenient for intervention. Through the second half of the last century the literature showed the connection between consciousness and illness[1,2,3,4,5,6,7,8,9,10,11,12,13,14,15,16,17,18,19,20,21]. David Spiegel et al.[21] showed that women with metastasised breast cancer lived twice as long if they improved their QoL. Self-perceived health has proven a surprisingly good health parameter predicting survival[7,8,10,11,16].

In our work with QoL we have focused on measuring global and generic QoL, using a self-administered, theory-based questionnaire. The factors mentioned above were sorted according to those that are influenced by man himself and those that are inherently inaccessible to the influence of the person himself. The first category contains factors such as the quality of personal relationships, which is greatly contingent on the attitudes and behavioural patterns of that person; personal philosophy of life; and processing the personal history. These factors all show an extensive connection to QoL and theoretically it should be possible to improve upon them, i.e., bring them in greater harmony with life, without changing the surrounding world. The last category is made up of annual income, level of education, and the quality of the environment.

The extensive connection between QoL and health is hypothesised to be causal from QoL to health[28]. This means that health improves when QoL improves. This hypothesis must be scientifically tested through intervention studies. The factors that were subject to influence were brought together in a concept for a 5-day seminar. Based on their personal philosophy of life, the therapists were carefully chosen so that their philosophy and understanding of illness was in keeping with the basic ideas of the 5-day cure.

## THE PHILOSOPHY BEHIND THE PROJECT

The idea behind the project was based on the idea that the human being exists in what is best described as three different inner worlds parallel to each other: a mental world, an emotional world, and a physical world. Each contains its own dimension of joy and pain, for instance, the joy of insight and the pain of confusion, positive and negative feelings towards others, and sexual satisfaction or frustration.

The three worlds — the mind, feelings, and the body — often live their own lives inside us and are normally taken as free to develop independently of each other. Training and teaching, e.g., in philosophy, can improve our understanding of life. When processing our personal history, therapy can help us towards a healthier emotional life and when we work with our bodies we become more present in them and "make the energies flow".

In reality, the three worlds are closely connected inside us, much more than we normally imagine. Our bodies, our feelings, and our attitudes and behaviour are really interwoven. When many people today suffer from physical and psychological illnesses, it is due to the lack of understanding between these connections.

The concept of "quality of life as medicine" was based on the simultaneous development of our philosophy of life, our emotions, and our presence in our bodies. Insight into life was improved analogous with an expansion of our emotional space and the dissolution of old blockages in the body. Experience showed that this leads to considerable improvement in QoL. The question is: is it possible to create a scientifically usable recipe for a psychosomatic cure?

The research project was built around three elements:

- **Philosophy of life.** Find your constructive philosophy of life. This gives the person a psychological framework for better self-realisation.
- **Psychotherapy.** Process your personal history. Psychotherapy is about integrating life events with negative emotional charge, turning psychological pain into understanding.
- **Body therapy**. Become present in your body. The body therapy can be any kind of bodywork that makes you once again aware of the areas in your body, which you have escaped throughout your personal history. The body therapy has the effect that it turns physical pain back into the original psychological pain, which then can be processed in psychotherapy.

QoL will be developed when attitude of life and life practise conforms to that recipe for being a human being, which we carry deep within ourselves, because by nature we were all created happy, full of vital energy and motivation as well as wise. Nature has equipped us with the knowledge of important issues in life, such as love, sex and cohabitation, friendship, upbringing of children, fellowship, personal and professional development.

When we bring these qualities forth we bring our whole organism into balance[5,20,22]. This inner balance is related to the information system of the organism, which means that the person who achieves an inner balance also achieves a sharper and clearer flow of information to his cells, and thus better health and well being. These ideas are the backbone of the philosophy of life that was presented to the participants at the seminar.

## PURPOSE OF THE STUDY

The purpose of the experiment was to show that self-perceived health, QoL, and working-life quality (QWL) were susceptible dimensions that could be improved considerably in a short time, and in the survey in just 5 days. It should also prove that perception of one's own health — like chronic pain, depression, or sleeping problems — can be improved when QoL is improved[23].

This study was a pilot study, but folow up has been planned to see if there are long-term effects of the seminar and to check if physical health also objectively follows QoL.

Finally, the purpose of the experiment was to qualitatively and quantitatively test the hypothesis that QoL and QWL can be improved effectively by a combination of philosophy of life, psychotherapy, and body therapy.

## METHODS

The study was an intervention study without a control group, but we compared the achieved results with data from 2,500 persons in the Danish QoL population survey[24]. The group of participants in the seminar was heterogeneous with regard to diagnoses. Common for the group was poor QoL, poor QWL, and many health problems.

The end points or dependant variables of the study were QoL, QWL, and self-perceived psychological and physical health, and satisfaction with health and the number and intensity of the health problems. The participants suffered from chronic pain that was considered incurable and outside the reach of traditional medical or therapeutic treatment, including pharmacology.

The participants were 19 long-term ill persons from the borough of Silkeborg in Denmark, who had been out of work for 5–7 years. They were part of a group of 40 people attending a rehabilitation project at the company Comtec in Silkeborg. The group could be characterized as "too good to be pensioned, but too poorly to work". They volunteered for the experiment after being introduced to the concept and the underlying philosophy of life with the main message that all people have large hidden resources to be used for improving life and health. Typical symptoms for participants were those connected with the diagnoses of fibromyalgia, chronic tiredness, whiplash, mild depressions, and problems involving pain in arms and legs including osteoarthritis.

The 19 participants were measured the week before and after the 5-day intervention seminar (response 95 and 90%, respectively). The self-administered questionnaire for "Self-Evaluation of Quality of Life" (SEQOL) was used, a questionnaire developed for the "Quality-of-Life" study at the University Hospital of Copenhagen[25] and supplemented with questionnaire on "Working-Life Quality"[26]. The questionnaires contained about 500 questions. A qualitative form containing open questions and textual responses was also used and the forms were completed in the usual surroundings of the participants.

The group was divided into two smaller groups that received therapy simultaneously. Mornings and afternoons were devoted to lectures on philosophy of life. Evenings were for individual sessions in psychotherapy and body therapy for those especially in need of this. Strangers, observers, or friends were not allowed into the therapy rooms while the philosophy room was videotaped for documentation. All participants were bound to secrecy regarding experiences of other participants. The environment for the seminar was at the Paradise scout camp in beautiful surroundings with neither radio nor television.

## MEASURING QUALITY OF LIFE

From 1991 to 1993, together with the Working Group for Quality-of-Life Research in Copenhagen, we prepared a summary of different views on QoL. It also became obvious to us that clear definitions of the concept of QoL together with good theories that can operationalise these definitions were necessary.

This gradually led to the following concept for measuring global, generic QoL[23], which today is being used by the Quality-of-Life Research Centre:

1. A clear definition of QoL.
2. A philosophy of life from which the definition is derived.
3. A theory that operationalises this philosophy by tracing questions that are unambiguous, mutually exclusive, and together fully able to cover, and determining the relative importance of each question.
4. Response possibilities that can be quantitatively interpreted on a fraction scale.
5. Technical checks (reproducibility, sensitivity, and suitable scale qualities).
6. The survey must have meaning for researchers, respondents, and those using the results.
7. Respect for the artistic dimension.

As explained in "Measuring the Quality-of-Life"[23] we have constructed a measuring scale (a "reduced combination scale") that goes from minimum QoL (0%) to maximum QoL (100%), which is an approximate ratio scale. For questions evaluating quality like "How satisfied are you with your health at present?" or "How good is your relationship with your partner?", we have divided the scale into five points (90, 70, 50, 30, and 10% of maximum), which corresponds to: "very good", "good", "neither good nor bad", "bad", and "very bad". For example, one of the goals of QoL is feeling good. If the response to the question "How are you feeling at the moment?" is "good", the respondent subjective quality of feeling good is estimated at being 70% of maximum. This is the number that is presented in the tables of the present work.

The other eight goals of QoL, e.g., satisfaction with life, are more complex because you really have to make a survey of your whole life[23,24,27].

Some important dimensions of QoL that were used in the survey measured by the SEQOL questionnaire[23,28]:

1. **Feeling good.** The most immediate aspect of subjective QoL can be called the quality of "feeling good"[23]. When we ask each other "How are you?", this is the aspect that needs a spontaneous, overall assessment. In reply we do not expect a deeper reflection because when we talk about how good we feel, we are usually quite a way from talking about how satisfied we really are with life or if our life has meaning. Therefore, feeling good is somewhat superficial compared to, for instance, satisfaction with and the meaning of life. Please note that we can tell everybody how we feel, but only a few are able to take stock of our meaning of life. So, we have a surface available for all to see and a hidden depth available to only a few, sometimes not even our own conscious self.
2. **Satisfaction with life.** When you ask people how satisfied they are with life, the answer usually is that something is wrong. Typically, people are less satisfied with life compared to how good they feel. There is always something not quite right when they think about it. To be satisfied means that life is as it should be. You have expectations, demands, and wishes in life and when you find that the world acts in accordance with these aspects you are satisfied. This can come about in two ways: you either try to change your surroundings so they fit your dreams, which then come true, or you give up your dreams as unrealistic and adapt to the world as it is, which also brings your surroundings and your dreams in concord. Both provide the same satisfaction, but it is obvious that these two life strategies provide for two quite different types of lives: the one fulfills your dreams, the other a life lived in resignation. Therefore, satisfaction with life does not necessarily mean a display of life or a need for fulfillment. However, you can be satisfied with life and deep down feel it is meaningless. It is quite common to be satisfied, but not happy.
3. **Happiness.** Most people will use this word only sparingly because it is treated with a certain respect. To be happy is not just being joyful or satisfied. It is a great and valuable state of mind, which is very attractive but difficult to achieve. Happiness is something that lies deep within people and it implies a certain balance or harmony. Happiness is intoxication; a rare sweetness of life, a murmur of little bubbles, and it is best described in pictures and preferably by poets. Happiness embraces the whole existence and is characterised by a certain intensity. In this way happiness is separate from the more superficial aspects of QoL such as satisfaction and feeling good, but they are all part of the subjective side of life.
4. **Fulfilment of needs.** This is the first of the four existential goals of QoL thus named, because they attempt to tap into the deeper aspects of life. Need is an expression of human nature, which means that you have a high QoL when your needs are fulfilled. Maslow's famous theory[14] of need assumes that man has needs not only of food, security, and social relations, but also needs to realise himself. This idea has penetrated our culture and has become intuitively meaningful. Thus the concept of need is nearly the closest we can get, linguistically, to the inner QoL.

5. **Family, work, leisure ("thriving").** The fifth goal of QoL takes a closer look at "feeling good" by asking questions about the three periods of time of the modern society: time at home with the family, time spent at work, and leisure time (days off, holidays, hobbies). This provides a subjective evaluation of an objective (culture-bound) division of life and consequently a goal for the time-divided life's deeper meaning.
6. **Satisfaction with things as they are.** Another way of tackling a meeting between the subjective and objective is to divide life into a number of objective "spacious" domains and ask for the subjective evaluation of these. We have divided life into five domains covering self, partner, children and parents, friends and acquaintances, and society and nature. The respondent states his degree of satisfaction with each of these domains: "How satisfied are you with your health?", "Your personal relationship?", "Society?" etc. The responses are weighted and a total goal of QoL, satisfaction with things as they are, is achieved.

## DEVELOPING QUALITY OF LIFE

The seminar on Quality-of-Life and Health came about by combining three kind of intervention: training in philosophy of life, psychotherapy, and body therapy. Some basic principles of these three are listed below.

### Philosophy of Life: Find Your Constructive Philosophy of Life[22,23]

1. **Reorientation towards life.** The good life is a life where we realise ourselves by knowing ourselves and our own wants, needs, resources, and potentials, and by knowing the possibilities in the world and the surroundings and using these to the full. Our hidden resources are acknowledged. The meaning of life is to make all our relationships good and fruitful — to create value for ourselves and our surrounding world.
2. **Identification of bad attitudes.** The problem we are struggling with is ourselves or, more precisely, all the bad attitudes we have acquired through our personal history of defeats and victories, confirmations and invalidations.
3. **Identification of inappropriate behaviour, which makes life what it is.** We make our own lives, but usually do not see it. Our consciousness is self-affirmative, which means that what we believe we always make happen in our reality whereupon we are confirmed in what we believe.
4. **Identification of better attitudes, which lead to a better life.** Life contains wisdom, joy, and vital energy, which can be acknowledged and expressed in a new belief in life and its possibilities, especially our own influence on our own existence.
5. **Identification of the new, constructive behaviour.** Better behaviour matches the new and better attitudes and forms life as better, more healthy, and positive.
6. **Decisions and consequent action on the new, better attitudes.** When we commit ourselves to practise better attitudes to life in our everyday lives, our lives change. Negative aspects are cleared from our personalities and we find a new freedom to flourish and develop.

### Psychotherapy

1. The good life is about creating and maintaining our own inner emotional space. In this space we build up our being, our self-esteem, our confidence, and our happiness.

2. A prerequisite for creating and maintaining your inner space is to know and respect the limits of this space. The art of living means defining our own limits both wisely and correctly — to delimit ourselves and see ourselves as separate from the surrounding world and other people, to simultaneously experience ourselves as being a harmonious part of the whole.
3. The difficulty in creating and maintaining the emotional space and its boundaries arises, because for years — our personal history — we have carried a lot of bad attitudes and correspondingly inappropriate behaviour around with us, which cause us to violate our own space and our own limits and allow others to offend us. When we let go of all the negative aspects we have collected through life from unfinished events in our personal history, life again has room to blossom within us.
4. Life with others is first and foremost about honest and respectful communication. This presupposes that we have our own inner space and limits in place.
5. With the ability to act without violating our inner space and limits and with the ability to communicate honestly and respectfully we can rebuild our lives. We can become free, living human beings who accept and trust other people without using force, i.e., without demanding, criticising, and controlling each other. The ability to communicate can improve gradually to encompass bigger and bigger parts of our surroundings and our personal universe.

### Body Therapy

1. All suffering is due to blockages of the body "vital energy", which again is connected to our lies and denials of life. When we let go of our physical tensions we release body energy and feelings.
2. When we touch areas on our body, we can easily feel the blockages, and when we work with ourselves, we can also discover from where in our personal history these blockages stem and the painful feelings and vital denials they are connected to.
3. QoL in the sense of physical well-being is about being present in all parts of the body. It is necessary to recapture the inner space of the body. Your breathing releases the old blockages and sets free the feelings that have been tied up in the body for a long time. A blockage is a regional withholding of breath and thus a withdrawal of our attention from the area.
4. Body parts are connected in a subtle system of "body energy": biological information, awareness, and purpose in life. When intuitively we have acknowledged this system, it is simple to move problems from the physical side to the psychological side. To put it another way, you can transfer the pain from being physical to being psychological after which you can confront it and get rid of it for good.
5. "Body energies" appear as emotional interpretations of the "stream of information" in the body. The body possesses a perfect knowledge of why it became ill and how to become well again. Therefore, the art of becoming well again is totally about learning to listen to the body.

## RESULTS

At the first measuring, the QoL of the participants was clearly below average compared to the Danish population at large[24]. The subjective "life experience" rated 62.9% as compared to a normal 70.0%. “Satisfaction” was also lower, 68.9% compared to 73.0% in other groups. “Needs” also rate a fraction below general average, 62.3% compared to 64.0%.

Compared to this, QoL at the second measuring was considerably higher than at the first measuring, and a little above the normal level. The subjective “life experience” was 76.9% as compared to a normal 70.0%. “Satisfaction” had also gone up, but still a little lower than normal, 75.5% compared to 73.0%. “Needs” increased from 62.3 to 76.5% at the second measuring and was above the general average of 64.0%.

Most movements between first and second measuring within the dimension QoL were found in “life experience” and fulfilment of “needs”, both of which increased by 14 percentage points.

## Health

The first measuring showed a considerable difference between the 19 participants and others Danes in general with regard to the dimension health[24]. The “total health” was assessed at merely 50.4% compared to the general level of 71.0%, a difference of nearly 21 percentage points.

“Physical health” rated 58.0% again clearly below the general average of 71.0%. The same applied to “psychological health”, where the 19 participants rated 59.3% compared to the average of 71.0%. “Satisfaction with health” was a low 47.1% compared to an average of 72.0%. Also the “absence of symptoms” was a problem compared to the general level, 31.8 and 63.0%, respectively. Days off due to sickness also showed the 19 participants at the bottom, 56.9% compared to 80.0% with 100% signifying no illness.

When comparing the second measure with the first, appreciable changes in the dimension “health” had taken place. The “total health” now rated 65,8%, an increase of about 15 percentage points. At the second measuring “physical health” rated 70.0%, an increase of 12 percentage points; “psychological health” showed 76.7%, an increase of 17 percentage points. “Satisfaction” with health increased by 21 percentage points and now rated 68.6%. “Absence of symptoms” was 56.7%, an increase of 25 percentage points. If first and second measuring is compared, it will be noted that the greatest improvement took place within the dimension “health”. The participants were feeling better, both physically and psychologically.

## Skills

Under “self-assessment of skills”, mastery rated a little higher, 66.4% compared to 62.0% in other groups. The “character of work” was also estimated a bit better than elsewhere, 66.6% compared to 65.0%. Also, “influence” was higher, 66.9% compared to 59.0%; and “experience of working”, 6l.6% compared to 62.0%. The participants’ “commitment to work” rated somewhat lower than that of other groups, 55.7% compared to 67.0%.

During the first measuring of the dimension “salary and status”, the 19 participants were well below the general average, 52.8% compared to 78.0%. “Development” was also well below average, 39.4% compared to 53.0%.

“Experience of working” increased to 69.1% compared to 61.6% at the first measuring. The “commitment to work” increased considerably at the second measuring to 64.4% and the response on “salary and status” rose to 59.3%, which is still far below the general average of 78.0%. The dimension “development” went up to 53.0%, which corresponded to the general level.

The greatest movements between first and second measuring within the dimension “commitment” increased by nearly 10 percentage points, and the dimension “development” went up with about 14 percentage points.

## Working Relationships ("Fellowship")

"Commitment" was at exactly the same level as that of others, i.e., 64.0%. "Information" was rated higher, 67.3% as compared to the general level of 57.0%. The dimension "teamwork" rated a little higher, 65.8% compared to 62.0%. "Working environment" was also higher, 68.7% compared to 63.0%. "Management" rated 70.5%, a little above the average 65.0%. "Relations" reached 77.3%, which was well above the general average of 67.0%. "Influence" also rated higher, 60.2% compared to 53.0%.

At the second measuring, "commitment" rose a fraction to 65.7%; "information" rated more or less at the same level, 68.0%. The dimension "teamwork" went up to 69.6%, "working environment" rated 7l.3%, "management" 72.7%, "relations" 76.7%, and finally "influence" rated a little lower, 57.1%.

The movements from first to second measuring were comparatively small in the dimension "fellowship".

## Creating Value

The "company's mission" rating was more or less like that of others', 70.4% compared to 70.0% in general. "Quality" rated a little lower, 63.3% compared to 65.0%. However, "efficiency" was a bit higher, 67.4% compared to the general level of 75.0%. The participants' "self-estimated ethics" rated quite a bit higher than others', 76.7% compared to the general level of 63.0%. "Creating value" was also higher compared to others as far as the participants were concerned, 66.1 and 59.0%, respectively.

At the second measuring the "company's mission" increased to 77.6%, "quality" to 71.3%, while "efficiency" rated 71.7%. "Self-estimated ethics" increased a fraction to 78.7% and the dimension "creating values" now reached 71.7%. The movements between first and second measuring of the dimension "creating value" was mainly an increase of 5–8 percentage points all round with the exception of "ethics", which only rated an increase of 2 percentage points.

## Summary of the Results

"QoL" rated somewhat below average, 65.0% compared to the normal level of 69.0%. "Mastery" also rated quite low, 58.4% compared to 65.0%. On the other hand, at the first measuring "fellowship" (working relationships) rated higher than average, 67.7% compared to 62.0%. Also "creating values" rated higher, 68,8% compared to the general level of 63.0%. The dimension "health", however, was the critical factor as the 19 participants at the first measuring reached as low as 50.4% compared to the normal level of 71.0%.

The second measure showed a number of movements towards the positive. "Self-evaluated QWL" had increased to 75.8%, an increase of approximately 12 percentage points. "QWL" now rated 71.3%, a modest increase of nearly 6 percentage points while "QoL" increased by 11 percentage points and at the second measuring rated 76.2%. "Mastery" increased by 8 percentage points to 66.3% while the dimension "fellowship" (working relationship) only had a moderate increase of about 1 percentage point to 68.7%. "Creating value" was now at 74.2%, an increase of about 5 percentage points.

The pilot study showed an 11.2% improvement in QoL, a 6.3 improvement in QWL, and a 12.0% improvement in self-perceived psychological health and satisfaction with health in general improved by 21.4%. Symptoms like pain were almost halved and several of the participants were free of pain for the first time in years.

"Self-assessed QWL" rated a little below average at the first measuring, 63.8% compared to 66.0%. The most interesting factor of the second measure was clearly the dimension "health", which increased from 50.4 to 65.8%, an increase of nearly 15 percentage points. Self-assessed "physical health" ($p = 0.08$), self-assessed "psychological health", self-assessed "QWL", and the measured QoL were appreciably and significantly improved by the seminar.

## DISCUSSION

The size of the changes in QoL and working life and health were unexpectedly large. Considering that only a 5-day seminar of intervention took place, the difference of 10–20% in the above mentioned dimensions must be seen as great and distinctive.

The gains of the participants are not likely to be permanent without reinforcement although it is expected that they will not return to their previous low level. Good attitudes are known to decay over time and must be reinforced. The new understanding of life and understanding of the basic principles of personal development, which the course provides to the participants, are thought to be permanent.

As this was a pilot study without a control group, the results are not final. The huge changes over a week against the background of years of chronically poor health and QoL for almost all participants were unlikely to be incidental.

The scientific method suffers from the lack of a control group because it is easy to suspect that just being removed from your everyday routine and placed together with others in a cabin in the woods for 5 days will make a great difference to your QoL.

The group, which medically speaking is badly defined, is another problem. On the other hand, apparently, the group was typical for people from the borough of Silkeborg who were too good to be pensioned, but too sick to work. The group was well known as chronically sick and abandoned by the traditional health system and family doctors.

The intervention came about through cooperation between the Quality-of-Life Research Centre and a private company, Comtec, who had applied to the European Union for funds to improve QoL, QWL, and health.

The great results from this study cannot be seen as a final expression of the value of the seminar or its concept. This is partly because the effect was expected to diminish with time and partly because a control group is needed, as well as more uniform diagnoses.

How you experience your health is not necessarily the same as changing an objective diagnosis. On the other hand, experience is what is essential and relevant for the patient and because of this, an improvement in QoL, QWL, and health is important and valuable also without objective improvements.

## CONCLUSION

The experiment showed an average improvement of QoL of 11.2%, an improvement of QWL of 6.3%, and an improvement of self-perceived "physical health" of 12.0% ($p = 0.08$). Self-perceived "psychological health" and "satisfaction with health" improved to 17.3 and 21.4%, respectively. A final documentation of the effectiveness of the cure is not available from this study, as it is a pilot study without a control group.

Apparently it is possible to improve QoL, QWL, and experience of and satisfaction with life with a 5-day cure. Chronic pain and other symptoms seemed to be reduced dramatically with the cure that combines philosophy of life, psychotherapy, and body therapy.

The great sensitivity that the subjective health dimensions have shown to changes in QoL is very indicative of psychosomatic reasons for problems with pain in arms and legs. Therefore, an unkind philosophy of life, an unprocessed personal history, and lack of presence in the body can also be important, contributing factors to pain and suffering.

The results from the study seemed to indicate that physical pain can be turned into psychological pain, which again can be converted into insight into life. This eliminates an inner conflict in the form of either a lie-about-life or self-refusal, which originated the pain. Popularly speaking, man can become free of illness and suffering when the soul is set free and he develops his true inwardness.

In conclusion it seemed that the combination of training in philosophy of life, psychotherapy, and body therapy can give patients a large, fast, and efficient improvement in QoL, QWL, and health. It is not known if these changes will be permanent and if all kinds of patients with different health problems will gain from this cure. Further research in this regard with control groups is being conducted.

## BIOSKETCH

**Søren Ventegodt, M.D.,** is the Director of the the Quality of Life Research Center in Copenhagen, Denmark. He is also responsible for a Research Clinic for Holistic Medicine in Copenhagen and is a popular speaker throughout Scandinavia. He has published numerous scientific, popular articles and a number of books on holistic medicine, quality of life, and quality of working life. His most important scientific contributions are the comprehensive SEQOL questionnaire, the very short QoL5 questionnaire, the integrated QoL theory, the holistic process theory, the life mission theory, and the Danish Quality of Life Research Study at the University Hospital of Copenhagen with the late pediatric Professor Bengt Zachau-Christiansen. E-mail: ventegodt@livskvalitet.org. Website: www.livskvalitet.org/

**Joav Merrick, M.D., D.MSc.,** is Professor of Child Health and Human Development affiliated with the Zusman Child Development Center and Division of Community Health at the Ben Gurion University, Beer-Sheva, Israel and presently the Medical Director of the Division for Mental Retardation, Ministry of Social Affairs, Jerusalem and the Director of the National Institute of Child Health and Human Development. He has numerous publications in the field of child and human development, rehabilitation, intellectual disability, disability, health, welfare, abuse, advocacy, and prevention. Dr. Merrick received the Peter Sabroe Child Award for outstanding work on behalf of Danish Children in 1985 and the International LEGO-Prize ("The Children's Nobel Prize") for an extraordinary contribution towards improvement in child welfare and well being in 1987. E-mail: jmerrick@internet-zahav.net. Website: www.nichd-israel.com

**Niels Jørgen Andersen, M.Sc.,** Professor, Department of Innovation and Economic Organization, Norwegian School of Management. This department conducts research and provides teaching in central topics related to innovation, business development, management of global companies, business history, and economic organization. Research activities within the Department are related to four core subjects within the discipline: business history, cooperative organizations, business development and entrepreneurship, and finally studies of industries with a special focus on the electricity industry. He is also the chairman of the board of the non-profit organisation Stiftelsen Holistisk Medisin Scandinavia, which works for the scientific development and documentaion of the holistic, complementary, and alternative medicine in the Scandinavian countries. E-mail: niels.j.andersen@bi.no. Website: www.bi.no/users/fgl93013/
